# Physical Activity in Fontan Patients Relates to Quality of Life and Sleep Quality

**DOI:** 10.3389/fcvm.2022.915810

**Published:** 2022-06-14

**Authors:** Alessia Callegari, Kathrin Faeth, Charlène Pfammatter, Ruedi Jung, Florian Berger, Barbara Burkhardt, Emanuela R. Valsangiacomo Buechel

**Affiliations:** ^1^University Children's Hospital Zurich, Pediatric Heart Centre, Zurich, Switzerland; ^2^Children's Research Centre, Zurich, Switzerland; ^3^University of Zurich, Zurich, Switzerland; ^4^Epidemiology, Biostatistics and Prevention Institute (EBPI), Zurich, Switzerland

**Keywords:** single ventricle, Fontan, physical activity, quality of life, sleep quality

## Abstract

**Background and Aim:**

Fontan patients tend to have reduced physical exercise capacity. This study investigates physical activity (PA) and its relationship to exercise capacity, heart rates, cardiac function, biomarkers, health-related quality of life (HRQoL), and sleep quality.

**Methods:**

Cardiovascular magnetic resonance (CMR), exercise testing (CPET), 24 h-ECG, and blood samples were prospectively performed in 38 patients, age 13 (11–16) years. PA was assessed by accelerometer during 7 consecutive days. HRQoL was self-assessed with KIDSCREEN-27 and SF-36 according to patients' age; sleep quality with Pediatric Sleep Questionnaire (PSQ) and Pittsburgh Sleep Quality Index (PSQI).

**Results:**

Daily moderate to vigorous physical activity (MVPA) was in median (IQR) 40 (28–57) mins; 7/38 (18%) patients reached the recommended 60 mins/day of MVPA. MVPA did not correlate with gender, age, single ventricle morphology, time from Fontan, heart rate, ventricular volumes, and ejection fraction at CMR, biomarkers, or CPET. Physical wellbeing (*r* = 0.33, *p* = 0.04), autonomy (*r* = 0.39, *p* = 0.03), and social support (*r* = 0.43, *p* = 0.009) assessed using the KIDSCREEN-27, and both physical (*r* = 0.57, *p* = 0.03) and mental (*r* = 0.54, *p* = 0.04) domains of the SF-36 questionnaire correlated with daily minutes of MVPA. PSQI global sleeping score (*r* = −0.7, *p* = 0.007), and PSQ scales for behavior (*r* = −0.36; *p* = 0.03) correlated with daily minutes of MVPA.

**Conclusion:**

Only 18% of the Fontan patients meet the recommendation for daily MVPA. Measures of exercise capacity, cardiac function or chronotropic competence are not correlated to daily physical activity. In contrast, HRQoL and sleep quality seem to be associated with regular physical activity.

## Introduction

Treatment of patients with a single ventricle consists of a staged procedure, the last step being the Fontan operation (1). In the last decades relevant developments in surgical, interventional, and medical therapies lead to a dramatical raise in survival rate of Fontan patients. Nevertheless because of the unphysiological hemodynamics of the Fontan circulation, morbidity remains important during mid- and long-term follow-up (1). It is well known that objective exercise capacity in Fontan patients is significantly decreased compared to normal subjects (2–5). The postulated reasons for this are various and include surgical factors, cardiac factors, such as systolic and diastolic dysfunction, morphology of the dominant ventricle, abnormal chronotropic response, diseased pulmonary vasculature, as well as lung dysfunction, cachexia, and decreased muscle mass (1). Guidelines for physical activity (PA) and sport participation in Fontan patients generally recommend light- to moderate intensity activity (6, 7). Some degree of PA is also recognize to help for peer and family socialization (8), reaching of normal developmental milestones, and decreasing the risk for sedentary lifestyles (9). Moreover, training programs for Fontan patients positively influence their objective exercise capacity (10) and self-reported as well as proxy-reported health-related quality of life (HRQoL) (10, 11).

In previous studies the reported PA level in Fontan patients failed to reach the current PA recommendations for the healthy population (2–5, 12–15). Only few authors have marginally assessed the effects of PA on quality of life (HRQoL) and sleep quality (11, 16).

The purpose of this study was to determine PA levels in a cohort of children, adolescents, and young adults with a Fontan physiology, and to focus on the relationship between PA and patient characteristics, cardiac function, exercise capacity, heart rates, serum biomarkers, and particularly HRQoL, and sleep quality.

## Methods

### Study Design and Patients' Selection

This is a single centre, prospective, observational study (2019–2021) in a cohort of 38 Fontan patients, children and young adults. The patients were identified from the cardiac electronic database of our institution. Inclusion criteria were ability to perform cardiopulmonary exercise testing (CPET) and to undergo cardiovascular magnetic resonance (CMR) without the need for sedation, as well as written informed consent of the subject or his/her legal guardian. Exclusion criteria were younger age, any contraindication for CMR or pregnancy. Every test was performed following the clinical standards of our institution. All tests were performed within a maximal time interval of 6 months to each other.

### Physical Activity

Physical activity was measured using an accelerometer (ActiGraph^®^ GT3X+, Pensacola, Florida, USA) for 7 consecutive days, during a regular school/work week and including both weekdays and weekends) (17). Participants were asked to wear the device 24 h/day on the right hip. Participants with a wear time more than 4 days and longer of 10 h/day were included in the study. Activity was analyzed during the timeframe from 06:00 am to 10:00 pm using the manufacturer's software (ActiLife 6.13.4). The time during which the patients wear or non-wear the accelerometer was calculated with an automatic algorithm as reported by Choi et al. (18). The accelerometer registered accelerations with a frequency of 100 Hz and an epoch lengh 60 s. The total vector magnitude in counts was calculated from the three axes of movement (horizontal, vertical, and diagonal). The activity intensity was analyzed and categorized in accordance with Evenson's Actigraph cut-off points (moderate intensity PA was defined as >2,296 counts/min and vigorous PA as >4,012 counts/min) (17, 19). Time spent in activity of a defined intensity (light, moderate, or vigorous) was provided by Actilife by summing minutes in a day where the count met the criterion for that intensity. Moderate to vigorous physical activity (MVPA) was categorized as inactive (<60 mins of MVPA per day) or active (≥60 mins of MVPA per day) according to the World Health Organization guidelines (20). Moderate physical activity corresponds to activities such as brisky walking or biking with a light effort. Vigorous physical activity corresponds to activities such as jogging, hiking, or playing a team sport (20). Normal values regarding PA in Fontan patients are lacking.

### Bioparameters

Ventricular volumes and function (ejection fraction) were measured by CMR using a 1.5 T scanner (Signa HDxt and MRI 450 or GE Signa Artist GE Medical Systems, Milwaukee, WI, USA) and a 32-channel phased array cardiac coil. Steady state free precession (SSFP) cine images were acquired in a horizontal and vertical long-axis plane, as well as in a short-axis plane covering the entire single ventricle. The SSFP parameters were as follows: retrospective cardiac gating, 40 phases/cardiac cycle, TE 1.5–1.8 ms, TR 2.8–3.1 ms, flip angle 45°, bandwidth 125 kHz, matrix 224 × 224, number of excitations 1, field of view 250–350, views per segment 4–10 according to heart rate. In-plane resolution was 1–1.5 mm and true temporal resolution was <25 ms.

Blood-sample for cardiac biomarkers were collected the same day as the CMR scan, aliquoted into freezer vials, and stored at −80°C. All samples were frozen and thawed only once before analysis. NTproBNP and growth differentiation factor 15 (GDF-15) were measured in plasma by an ISO 15189 accredited diagnostic laboratory using a Cobas e411 analyser according to the manufacturer's instructions using commercial reagents (Roche, Rotkreuz, Switzerland) with coefficients of variation of 1.1% (139 ng/L) and 3.1% (1,322 ng/L), respectively. Protein delta homolog 1 (DLK-1) and insulin-like growth factor-binding protein 7 (IGFBP-7) were measured by ELISA in serum and were analyzed on a Synergy HT Multi-Detection Microplate Reader (BioTek, Winooski, VT, USA) according to the manufacturer's instructions with coefficients of variation in our samples (*n* = 5) of 13.8% (34.5 μg/L) and 10.5% (57 μg/L) respectively. These biomarkers were chosen based on previous data of the literature for (21–23).

24 h ECG and accelerometer registrations were started the same day of the CMR scan. Cardiopulmonary exercise test was performed on a cycle ergometer with a ramp protocol in accordance with our institutional standards (24). Resting parameters were defined as the mean values of the raw data acquired during 3 mins in a sitting position without cycling; the peak parameters were the mean values of the raw data acquired during the 30-sec time interval at maximal exercise (peakVO_2_).

### Health-Related Quality of Life and Sleep Quality

In patients older than 16 years the self-perceived HRQoL was evaluated using the questionnaire 36-item short-form (SF-36) The SF-36 questionnaire covers physical, mental and social aspects using eight categories of questions with scores ranging from 0 (bad) to 100 (best) and subscales with 2 to 10 entries, and has been validated in various categories of patients (25). Reference values for the normal adult population have been published (26).

Children younger than 16 years completed the KIDSCREEN-27 test, which is a short version of the original KIDSCREEN-52, together with their parents or guardians. The 27-item questionnaire assesses 5 HRQoL dimensions: Physical wellbeing, psychological wellbeing, arent relations and autonomy, social support and peers, and school. Each item is scored on a 5-point scale (from 1 = “not at all” to 5 = “very much”). For each dimension, a scoring algorithm is used to calculate “T-scores” scaled with reference values of 50±10 (27).

Evaluation of sleep quality was performed in children <16 years of age with a Pediatric Sleep Questionnaire (PSQ) (27), with their parents being asked to complete the questionnaire. The total sleep-related breathing disorder (SRBD) scale evaluates symptoms such as snoring, apneas, difficulty breathing during sleep, daytime sleepiness, inattentive or hyperactive behavior. The SRBD scale is divided in 3 subscales: 4-items for *Sleepiness*, 4-items for *Snoring*, and 6-items for *Attention/hyperactivity*. Every positive answer gives one point and a positive PSQ scoring; the cut-off for each sub-scale is 0.33, with a mean score of ≥0.33 being considered positive and a mean score <0.33 negative.

Patients older than 16 years completed the Pittsburgh Sleep Quality Index (PSQI) (28). This is a self-rated questionnaire which assesses sleep quality and disturbances over a 1-month time interval. The sum of 7 component scores results in a global score and the cut-off for pathology is set by 5 points. The German-language versions were used for all instruments.

### Statistics and Ethics

Statistical analysis was performed using SPSS 27.0.0 (SPSS Inc, IBM Company, Chicago Illinois/USA). Continuous variables are expressed as median (IQR), categorical data as counts (percentages). For normally distributed continuous variables, Levene's test for equality of variance was used to analyse if the variability in the two groups was significantly different and group comparison of was performed using two-sample *t*-tests. Kolmogorov-Smirnov analyses were used for group comparisons of non-normally distributed continuous variables. Ordinal, nominal, and dichotomic variables were evaluated with contingency tables and chi-square-tests. Correlations between continuous variables were tested using Pearson's (for the normally distributed variables) or Spearman's (for the non-normally distributed variables) correlations. Binomial logistic regression was used for dichotomic variables. Significance was defined by values of p < 0.05.

The study followed the ethical guidelines of the declaration of Helsinki for medical research involving human subjects. The study was approved by the local ethical authorities (KEK: 2018-01878).

## Results

### Patients and Cardiac Parameters

A total of 38 Fontan patients, 15 (39%) females, were included in the study. Median (IQR) age was 13.1 (11.0–16.2) years, weight 46.5 (32.3–60.6) kg, height 152 (140–166) cm, and BMI 18.3 (17.4–21.6) kg/m^2^. Twenty (53%) patients had a single right ventricle, being hypoplastic left heart syndrome the most frequent diagnosis (13 patients, 34%of all patients). Patients cardiac anatomies are presented in Table 1. Age at Fontan surgery was 2.6 (2.2–2.9) years and time interval from Fontan surgery 10.9 (8.3–12.2) years. The surgical technique of total cavopulmonary connection consisted of an extracardiac tunnel in all patients. Cardiac medication was taken by 19 patients (50%) and consisted of an ACE-inhibitor in 9 (23%), diuretics in 6 (15%) and sildenafil in 4 (10%) sildenafil. Anticoagulation consisted of aspirin in 35 (92%) and warfarin in three (7%) patients.

**Table 1:** Patients' characteristics.

**Cardiac anatomy**	***n***	**%**
Left single ventricle	18	47
Double inlet left ventricle	8	21
Tricuspid atresia	4	10
Pulmonary atresia with intact ventricular septum	3	8
Dysbalanced atrioventricular septal defect	3	8
Right single ventricle	20	53
Hypoplastic left heart syndrome complex	13	34
Double outlet right ventricle	6	16
Dysbalanced atrioventricular septal defect	1	3

At time of the study, two (5%) patients were treated for protein loosing enteropathy, and one (2.5%) for plastic bronchitis. Eight (21%) patients had a previous history of chylothorax, CMR measurements of ventricular volumes resulted in end-diastolic volume of 116.0 (81.7–136.0) ml/m^2^ and end-systolic volume of 51.5 (31.5–66.7) ml/m^2^. Median ejection fraction (EF) was 51.2 % (48.6–58.6), with 15 patients (39%) having an EF < 50%.

Serum cardiac biomarkers showed following values: GDF-15 502.2 (450.9–569.3) pg/mL, DLK-1 1.16 (0.41–2.82) ng/mL, IGFBP-7 101.4 (80.9–117.6) ng/mL, and NT-pro-BNP 59 (37–126) ng/L. NT-pro-BNP was within the range of normality, while for the other parameters validated normal values do not exist yet.

Median heart rate (HR) measured during 24 h-ECG was 80 bpm (75–89), maximal HR was 149 bpm (137–164). A sinus or atrial rhythm was present in 31 patients (79%), a combination of sinus and junctional rhythm in 4 (10%), and solely junctional rhythm in 3 (8%). A history of supraventricular arrhythmias was reported in 4 (10%) patients, but none of them was under antiarrhythmic medication at time of the study.

CPET showed, as expected, reduced exercise capacity with a peakVO_2_ of 28.7 (25.3–31.7) ml/kg/min. Maximal HR was 169 bpm (158–181) and maximal work was 2.1 (2.0–2.7) Watt/kg.

### Physical Activity

**Figure 1:**
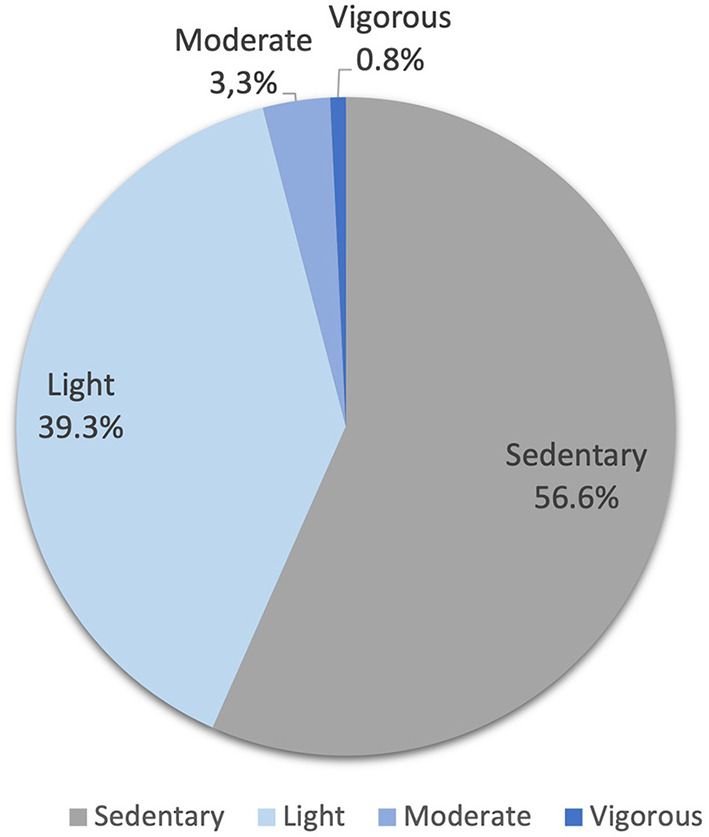
Categories of physical activity (percentage of time) during 7 days.

Levels and duration of PA are summarized in Table 2. The respective percentage (%) of light, moderate, and vigorous PA are shown in Figure 1. Overall the patients were sedentary during 56.6 (50.2–63.4) % of the time. Only 7 (18%) patients reached the cut-off of 60 min/day of MVPA for being defined as “active” according to the WHO guidelines. MVPA was registered in only 4.3 % of the total time (7 days), which corresponded to an average of 40 (29–55) min/day.

**Table 2:** Physical activity levels of all patients.

**Physical activity (min/7 days)**	**Median (IQR)**
Light	2,287 (1,761–2,568)
Moderate	177 (140–246)
Vigorous	47 (31.5–81)
**Physical activity, total of 7 days**	
Sedentary (%)	56.6 (50.2–63.4)
Light (%)	39.3 (32.9–43.7)
Moderate (%)	3.3 (2.3–4.6)
Vigorous (%)	0.76 (0.53–1.51)
**Moderate-to-vigorous physical activity**	
Total, 7 days (minutes)	249 (188–323)
Total, 7 days (%)	4.3 (3.4–6.2)
Minutes/day	40 (29–55)
> 60 mins/day, *n* (%)	7 (18)

### Health-Related Quality of Life and Sleep Quality

The KIDSCREEN-27 questionnaire was completed by 31 pediatric patients and the SF-36 test by 7 young adults. The results of the KIDSCREEN-27 (T-scores) and SF-36 (%predicted) tests are summarized in Table 3. Overall, the patients self-reported a good HRQoL. In the KIDSCREEN only the T-scores for the category physical wellbeing and social support & peers were lower than the expected value of 50. In the SF-36 vitality was in median only 61.7% of the expected value, while general health, physical functioning, and mental health were only slightly below the norm of 100%.

**Table 3:** Results of the SF-36 and KIDSCREEN-27 questionnaires and correlation with MVPA/day.

**Test**	**Score**	**MVPA/day**	
**KIDSCREEN-27 (*****n*** **=** **31)**	***T*** **-scores**	***p***	**r**
Physical wellbeing	47.08 (42.53–52.43)	**0.05**	0.33
Psychological wellbeing	53.07 (46.53–59.51)	0.30	0.18
Parent relations & autonomy	53.25 (49.47–56.58)	**0.03**	0.37
Social support & peers	49.79 (44.40–53.23)	**0.01**	0.44
School environment	54.40 (48.09–58.16)	0.08	0.29
**Questionnaire SF-36 (*****n*** **=** **7)**	**% predicted**	***p***	**r**
Physical functioning	93.94 (73.06–94.45)	**0.04**	0.56
Physical role functioning	101.74 (78.48–101.74)	**0.05**	0.53
Bodily pain	109 (109–109)	0.16	0.40
General health	91.42 (74.74–99.65)	**0.03**	0.58
Vitality	61.73 (34.29–96.02)	**0.04**	0.55
Social role functioning	106.18 (99.54–106.18)	0.08	0.48
Emotional role functioning	105.36 (70.24–105.36)	0.08	0.49
Mental health	93.96 (84.73–100.33)	**0.04**	0.56
*Component summaries:*			
Physical	93.96 (84.73–100.33)	**0.03**	0.57
Mental	107.45 (96.76–110.28)	**0.04**	0.55
Total	96.21 (88.89–102.23)	0.07	0.49

*Statistically significant correlations are marked in bold*.

Thirty-one children and their parents completed PSQ and 7 adults the PSQI (Table 4).

**Table 4:** Results of the PSQ and PSQI questionnaires.

**Test**	**Score**	**Patients**	**MVPA/day**	
**PSQ (*****n*** **=** **31)**		**>0.33**	**p**	**r**
Obstructive sleep-related breathing disorders	0.21 (0.13–0.47)	12 (39)	0.13	0.26
Subscale for snoring	0 (0–0.33)	4 (13)	0.06	0.50
Subscale for sleepiness	0.25 (0–0.5)	10 (3)	0.06	0.32
Subscale for inattentive/hyperactive behavior	0.33 (0.17–0.67)	17 (55)	**0.03**	**−0.36**
**PSQI (*****n*** **=** **7)**		**>5**	**p**	**r**
Global score	4 (2.25–5.57)	4 (57)	**0.01**	−0.71

*PSQ, Pediatric sleep questionnaire; PSQI, Pittsburgh Sleep Quality Index. The results of expressed as median (IQR) or n (%), as appropriate. The statistically significant correlations are marked in bold*.

The total PSQ score for obstructive sleep-related breathing disorders, as well as the subscale for snoring and that for sleepiness were in the normal range, i.e., <0.33. Nevertheless high scores (>0.33) were found in 12 (39%) patients for the total score, in four (13%) for snoring and in 10 (32%) for sleepiness.

The overall score for inattentive / hyperactive behavior was borderline with a value of 0.33 (0.17–0.67). Correspondingly, a score > 0.33 was obtained in 17 (55%) patients.

Similarly the overall global PSQI score for sleep quality and disturbances was normal, but a pathologic high scorer resulted in 4 (57%) patients.

### Predictors of Physical Activity Levels

No significant correlation was found between daily minutes of MVPA were and gender, age, BMI, single ventricle morphology, age at Fontan operation, time from Fontan operation, ventricular volumes and ejection fraction, serum biomarkers, heart rate and cardiac rhythm, CPET parameters, cardiac medication or any complications and/or sequelae.

### Physical Activity Levels and Quality of Life and Sleep

Daily minutes of MVPA showed a positive correlation with three sub-classes of the KIDSCREEN-27, namely physical wellbeing (*p* = 0.05, *r* = 0.33), parent relations & autonomy (*p* = 0.03, *r* = 0.37), and social support & peers (*p* = 0.01, *r* = 0.44). The SF-36 questionnaire showed a positive correlation between daily minutes of MVPA and five categories of both, physical health and mental health, as shown in Table 3.

Daily minutes of MVPA correlated with lower PSQI global score (*p* = 0.01, *r* = −0.71), and better scores for the subscale for inattentive/hyperactive behavior of the PSQ (*p* = 0.03, *r* = −0.36).

## Discussion

In this study we have assessed the physical activity measured by accelerometer in 38 young Fontan patients and its relationship to somatic and cardiac parameters, as well as its influence on HRQoL and sleep quality. In addition to other published data on PA in Fontan patients (2–5, 12–15), our results add information about the interaction with novel serum biomarkers and the influence of PA on HRQol and sleep quality.

### Physical Activity and Somatic Parameters

Notably, the median MVPA value measured in our cohort was 40 (29–55) min/day, and only 7 (18%) patients achieved a MVPA ≥ 60 min/day, defined by the WHO as a sufficiently active life style (12). In the largest cohort published so far, McCrindle et al. studied the PA level in 108 children and adolescents (7–18 years) and found that only 38% of the subjects achieved the age-specific physical activity recommendations (4). Similar findings than ours have been described by other authors with daily MVPA of 42 and 50 min/day respectively (2, 3). In contrast Hedlund et al. in a similar population and same accelerometer technique than in our study, reported an average MVPA of 148 min/day and 38% of the patients reaching a MVPA ≥ 60min/day (15). Most interestingly no difference was observed between Fontan teenagers and healthy teenagers in two studies with an internal age-matched control group (3, 15). Thus, the review of the literature suggests that most Fontan patients, but not all, present with reduced levels of physical activity. The reasons for lower PA remain unclear. We could not identify any clinical factors related to decreased PA levels. One may question if accelerometery is an ideal method for assessing PA levels in Fontan patients, even though activity measurement by accelerometer is a wellestablished and validated method in the pediatric population. One additional question can be if the WHO guidelines are an appropriate benchmark for patients with an unphysiological circulation. Our results and these considerations can be helpful for the design of future prospective studies.

Our results did not show any significant decrease in MVPA with increasing age, as it has been described by others (2, 4, 5, 15). The reasons for this can probably be found in the size and age distribution of the study groups (2, 4, 5, 15). A gender difference has been described by McCrindle et al. but not by others (2, 4, 5). We did not identified any anatomical or surgical characteristics affecting PA levels, nor did other authors for ventricular morphology (14, 15), time interval from Fontan completion (14), and/or type of Fontan connection (4, 5). Some have reported that a diagnosis different than hypoplastic left heart syndrome (4), older age at Fontan completion (2, 14), and fewer procedures prior to Fontan were related to higher PA levels.

Serum cardiac biomarkers have been associated with reduced objective exercise capacity and poor clinical outcome in Fontan patients (21–23). This is the first study testing novel biomarkers, such as GDF-15, DLK-1 and IGFBP-7, in relation to PA, and we did not observe any correlation. Similarly to Müller et al. we also did not find any correlation between NT-pro-BNP and PA levels (5).

It is well known that severe cardiac abnormalities have a major impact on objective exercise capacity (12). Some authors have also shown that patients with a higher exercise capacity have a more active daily life measured by accelerometer (3, 5, 12). Surprisingly we did not find any correlation between CPET parameters and MVPA. Interestingly, McCrindle et al. (4) in their larger patients cohort also described a lack of correlation between PA and objective exercise capacity. In this context we believe that several other factors may influence the time spent doing physical exercise in Fontan patients; these may include an extremely cautious medical counseling regarding selected sports (particularly in patients receiving anticoagulation), which may be understood as general dissuasion from sport by certain families, anxious parents blocking their children from sportive activities, patients self-perception and subjectively reduced exercise capacity (in comparison to peers) yielding to a behavior avoiding frustration, and lack of personal motivation (2–5, 10). All these factors/ behaviors can be influenced by cultural, socio-economic and familial structure, as well as geographical factors. Parental overprotection has been recognized to be an important issue for exercise limitation, as 50% of patients with congenital heart disease reported that their parents limit their physical activities (11). Reduced self-efficacy, particularly in adolescents and young adults, may also impact their degree of participation in PA (29). The reported decrease of PA with increasing age and the lack of difference between Fontan patients and control adolescents reported in some studies (3, 15) may question the concept of a fix cut-off for definition of ideal MVPA over the ages.

Recent reviews on structured exercise training in Fontan patients have described how regular physical activity is safe and beneficial for these patients. However it is important to note that punctual short-term interventions like a time-limited training program did not lead to a permanent improvement of MVPA (11, 30). These observations suggest that interventions aiming to improve PA levels should be established early during treatment of single ventricle lesions and be supported by postsurgical rehabilitation programs.

### Health-Related Quality of Life

As survival of single ventricle patients keeps improving, their quality of life represents an important goal to be targeted in the long term. Our study confirms a good HRQoL reported by the majority of Fontan patients (31). We have demonstrated that higher PA levels were associated with higher scores in many categories of both, the self-reported SF-36 and parents-reported KIDSCREEN-27. Interestingly, in the perceived HRQoL PA levels not only influenced the plain physical sphere, but also the general perception of health status and social wellbeing, which included parents relation and social support and peers in children, and mental health in adolescents and young adults. Few other authors also reported a positive correlation between PA levels and the self-reported psychosocial functioning and general health (4, 5). In contrast other studies did not observe any association between PA levels and parent-reported HRQoL (32, 33). Even though data are not completely consistent, our results suggests that specific interventions targeted to increase PA levels may have some beneficial effects not only on physical aspects but also on peer and family socialization and patients cognitive development (8, 9).

### Sleep Quality

In Fontan patients sleep disturbances have been related to a reduced HRQoL and neurodevelopmental impairment (34). Our patients reported sleep disturbances and inattentive or hyperactive behavior in up to 30–57%, depending on the specific item. Daily minutes of MVPA were correlated with a lower incidence of sleep disturbances, similarly as it was described by Hedlund et al. (16). Therefore, PA may have a positive effect on sleep quality and reduce hyperactive behavior in Fontan patients.

## Limitation

As a single center study on a rare cardiac condition, our results report on a limited number of subject, and statistical analysis may present some limitations. Moreover, the cohort may have a patients selection bias regarding age of the patients and comorbidity factors. We included children from an age, in which they were able to perform CPET and CMR without need for sedation; therefore, we could not cover the full age spectrum starting just after Fontan completion. Patients with a pacemaker have not been included because of the contraindication for undergo CMR. We have not included a control population; this would have provided the study with a valid group comparison for PA levels. Moreover, an appropriate use of the accelerometer could not be monitorized, as this was based on a short introduction and on reciprocal trust; some degree of monitor tampering cannot be completely excluded. The data acquired did not allow for analysis of the influence of familiar, saisonal, geographical factors or medical advice on the effective PA of the patients (2, 4, 14).

## Conclusions

Only a minority of Fontan patients (18%) meets the recommendation for daily physical activity. Measures of exercise capacity, cardiac function, or chronotropic competence are not correlated to daily MVPA. In contrast levels of PA are related to quality of life, not only in the physical but also in the mental domains.

## Data Availability Statement

The raw data supporting the conclusions of this article will be made available by the authors, without undue reservation.

## Ethics Statement

The studies involving human participants were reviewed and approved by Kantonale Ethikkommission Kanton Zurich, Switzerland. Written informed consent to participate in this study was provided by the participants' legal guardian/next of kin. Written informed consent was obtained from the individual(s), and minor(s)' legal guardian/next of kin, for the publication of any potentially identifiable images or data included in this article.

## Author Contributions

AC designed the study, was primarily involved in data acquisition, and data analysis and drafted the manuscript. KF and CP was involved in data acquisition and data analysis. RJ provided knowledge and technical support in data acquisition (accelerometer) and data analysis. FB and BB was involved in study design, data acquisition, and data analysis. EV designed the study, was involved in data analysis, drafting, reviewing, and revising the manuscript. All authors reviewed the manuscript.

## Conflict of Interest

The authors declare that the research was conducted in the absence of any commercial or financial relationships that could be construed as a potential conflict of interest.

## Publisher's Note

All claims expressed in this article are solely those of the authors and do not necessarily represent those of their affiliated organizations, or those of the publisher, the editors and the reviewers. Any product that may be evaluated in this article, or claim that may be made by its manufacturer, is not guaranteed or endorsed by the publisher.
